# GNE Is Involved in the Early Development of Skeletal and Cardiac Muscle

**DOI:** 10.1371/journal.pone.0021389

**Published:** 2011-06-24

**Authors:** Irit Milman Krentsis, Ilan Sela, Rachel Eiges, Véronique Blanchard, Markus Berger, Michal Becker Cohen, Stella Mitrani-Rosenbaum

**Affiliations:** 1 Goldyne Savad Institute for Gene Therapy, Hadassah Hebrew University Medical Center, Jerusalem, Israel; 2 Stem Cell Research Laboratory, Medical Genetics Institute, Shaare Zedek Medical Center, Jerusalem, Israel; 3 Glycodesign and Glycoanalytics, Institute of Laboratory Medicine, Clinical Chemistry and Pathobiochemistry, Charité, Berlin, Germany; Instituto de Ciencia de Materiales de Madrid - Instituto de Biomedicina de Valencia, Spain

## Abstract

UDP-N-acetylglucosamine 2 epimerase/N-acetylmannosamime kinase (GNE) is a bifunctional enzyme which catalyzes the two key sequential steps in the biosynthetic pathway of sialic acid, the most abundant terminal monosaccharide on glycoconjugates of eukaryotic cells. GNE knock out (GNE KO) mice are embryonically lethal at day E8.5. Although the role of GNE in the sialic pathway has been well established as well as the importance of sialylation in many diverse biological pathways, less is known about the involvement of GNE in muscle development. To address this issue we have studied the role of GNE during *in vitro* embryogenesis by comparing the developmental profile in culture of embryonic stem cells (ES) from wild type and from GNE KO E3.5 mice embryos, during 45 days. Neuronal cells appeared rarely in GNE KO ES cultures and did not reach an advanced differentiated stage. Although primary cardiac cells appeared at the same time in both normal and GNE KO ES cultures, GNE KO cardiac cells degraded very soon and their beating capacity decayed rapidly. Furthermore very rare skeletal muscle committed cells were detected in the GNE KO ES cultures at any stage of differentiation, as assessed by analysis of the expression of either Pax7, MyoD and MyHC markers. Beyond the supporting evidence that GNE plays an important role in neuronal cell and brain development, these results show that GNE is strongly involved in cardiac tissue and skeletal muscle early survival and organization. These findings could open new avenues in the understanding of muscle function mechanisms in health and in disease.

## Introduction

UDP-N-acetylglucosamine 2 epimerase/N-acetylmannosamime kinase (GNE) is a bifunctional enzyme with 2 distinct domains which catalyze the two key sequential steps in the biosynthetic pathway of sialic acid: the epimerase activity synthesizes ManNAc from UDP-GlcNAc, followed by ManNAc kinase phosphorylation of ManNAc to generate ManNAc 6-phosphate [1], [2]. Three subsequent steps lead to the synthesis of the active form of sialic acid, CMP-sialic acid, the donor of sialic acid to the terminal sugar in a glycan chain.

Sialic acid is the most abundant terminal monosaccharide on glycoconjugates of eukaryotic cells [3], and as such is involved in several biological pathways that are crucial in most cells and tissues. GNE is essential for embryonic development: specific knock-out inactivation of the gene in mice results in drastic reduction of sialylation of embryonal cells and in embryonal lethality at day E8.5 [4]. GNE is regulated by several different mechanisms, most importantly the feed-back inhibition of the epimerase activity by CMP-sialic acid, the precursor of the final product sialic acid [5]. Mutations in GNE result in 2 pathological manifestations: the first is caused by a missense mutation in the allosteric binding site for CMP-sialic acid, preventing its binding thus leading to the very rare dominant metabolic disease sialuria, characterized by highly abundant production and secretion of sialic acid by the patients [6]; the second, hereditary inclusion body myopathy (HIBM), is a unique recessive neuromuscular disorder characterized by adult-onset, slowly progressive distal and proximal muscle weakness, resulting from homozygous or compound heterozygous mutations that can occur both at the epimerase domain, both at the kinase domain, or one in each domain of the protein [7]. Marked GNE deficiency has not been observed in HIBM patients, in fact western blots show that the GNE protein is expressed at equal levels in HIBM patients and normal control subjects [8]. Furthermore, no mislocalization of GNE in skeletal muscle could be documented [8], [9]. However the enzymatic activity of GNE is reduced by about 30% [10]–[11] but it is not clear whether this reduction affects the biosynthesis of sialic acid: analysis of muscle cells from patients carrying the Middle Eastern founder homozygous mutation M712T, at the kinase domain of the enzyme, revealed a broad physiological range of bound sialic acid levels overlapping with the same broad range in normal controls [10], [11]. Some reports however documented sialic acid reduction in some patients with different mutations in GNE [12], [13].

Interestingly, an overall reduction of 25% in membrane bound sialic acid was observed in various organs of heterozygous GNE knock out mice [14]. In spite of these biochemical findings those mice were perfectly healthy and did not develop myopathy or any other pathological condition, even after 2 years. Thus, the process by which mutations in GNE lead to the disease is not yet understood, and the issue of hyposialylation in HIBM muscles is still unresolved [10]–[14].

Therefore we and others have hypothesized that GNE plays additional roles that could eventually explain the relatively mild and skeletal muscle specific phenotype of HIBM. Knocking out the GNE gene in mice results in mouse embryos lethality at day E8.5 and therefore these mice cannot be used as a tool for our purpose, although the cause of death has not been determined.

In an attempt to unravel novel role(s) of GNE in normal cells, we have used the embryonic stem cells from wild type and GNE−/− mice embryos, to study the differentiation patterns of these cells during very early development, to various tissues, in particular skeletal and cardiac muscle.

## Materials and Methods

### ES cells culture and differentiation

Embryonic stem cells were the kind gift of Dr Ruediger Horstkorte (Halle, Germany). GNE−/− cells had been fully characterized as having no GNE enzymatic activity [4], [15].

Cultures were established essentially as described by Wobus A. et al [16]: ES cells were thawed and cultured on an irradiated MEFs (mouse embryonic feeders) layer in Dulbecco's modified Eagle's medium (DMEM) (Sigma, Germany) supplemented with 15% heat-inactivated fetal calf serum, 2 mM L-glutamine, 50 U Penicilline/Streptomycine, 0.1 M nonessential amino acids (Beit Haemek, Israel), 5×10^−5^ M β-mercaptoethanol )Bio-Rad, USA) and with 5×10^5^ units of recombinant human leukemia inhibitory factor (LIF)/500 ml.

For differentiation, cells were grown in hanging drops to allow the formation of embryoid bodies (EB). On day 3, 1% of DMSO (Fluka, Switzerland) was added to the medium in order to favor skeletal muscle specific differentiation and EBs were grown in suspension for 3 more days. At day 5, EBs were plated on gelatin coated tissue culture plates for further differentiation. On average, 2 EBs were plated per well of a 6 well plate. For immunofluorescence, EBs were plated on LAB-TEC chambers slides (Nunc, Roskilde, Denmark). Cells were kept in culture up to 45 days and examined at different time points for morphology and specific markers expression. Time point 0 refers to proliferating ES cells before EB formation as a hanging drop. Day 5 refers to the plating day of EBs onto gelatin coated tissue culture plates.

### Expression analysis of specific markers by RT-PCR analysis

RNA was extracted from 2 wells of the 6 wells plate, with Tri-Reagent (Sigma, St. Louis, MO, USA.) at each time point, both from GNE KO and WT ES differentiated cells, as described by the manufacturer. RNA was reverse transcribed using random hexamer primers (Roche, Germany) by the Masterscript reverse transcriptase enzyme (5 prime, UK) following the protocol supplied by the manufacturer. The cDNA products were denatured for 5 min at 95°C, followed by 40 cycles of amplification with the appropriate primers: 45 sec denaturation at 95°C, 30 sec annealing at 55 to 72°C (depending on the primer used), and 30–120 sec elongation at 72°C followed by a final elongation at 72°C for 5 min. For β-tubulin detection, 30 cycles of amplification were performed and 25 cycles for NFM. One-fifth of each RT–PCR reaction was electrophoretically separated on 2% agarose gels. For MyoR detection the 7500 real-time PCR system (Applied Biosystems, UK) was used, together with mouse GAPDH, using power SYBR green master mix (Applied Biosystems, UK). The expression of the following genes was studied: Oct4, NFM, βMHC, Pax7, MyoD, MyoR (primer sequences are given in Table S1). β tubulin and GAPDH were used for normalization.

### Immunofluorescence staining

Cells were grown in LAB-TEC chamber slides (Nunc, Roskilde, Denmark), permeabilized and fixed with ice-cold methanol (15 min at −20°C). Cells were blocked in 2% BSA/PBS for 60 min at RT and incubated for 30 min at RT with mouse monoclonal anti Myosin Heavy Chain antibody (MF-20 hybridoma) (1∶5, 54 µg/ml; Developmental Studies Hybridoma Bank, University of Iowa.) and mouse monoclonal anti desmin antibody ( 1∶150, Dako) diluted in 2% BSA/PBS. After washing in PBS, cells were incubated 30 min at RT with goat anti-mouse Cy3-conjugated antibody (1∶200; Jackson ImmunoResearch) diluted in 1% BSA/PBS. Nuclei were stained by Hoechst (15 min, R.T., 10 µg/ml; SIGMA).

### Microscopic analysis

Light microscope pictures were viewed in CKX41 system inverted microscope (OLYMPUS, Tokyo, Japan),and photographed by a C5060 digital camera (Olympus). Immunofluorescence was viewed in a BX41 system microscope (UPlanFL N 40×/0.75 ,Olympus), photographed by a DP70 microscope digital camera and analyzed by the DP software (Olympus).

### Glycan analysis

For glycan analyses, cell pellets were dissolved in 0.2% SDS, 1% beta-mercaptoethanol, 0.1% NP40 and homogeneized.

#### Sialic acid determination

WT and GNE ko stem cell samples were subjected to sialic acid analysis [17]. Sample aliquots were hydrolyzed in 3 M acetic acid for 3 h at 80°C. After the reaction, samples were neutralized with ammonia, then evaporated. The internal standard, 2-keto-3-deoxy-nonulosonic acid (KDN), was added and sialic acids were specifically labeled with 1,2-diamino-4,5-methylene dioxybenzene (DMB, Sigma) for 2.5 h at 56°C using a solution containing 7 mM DMB, 18 mM sodium disulfite, 0.75 M b-mercaptoethanol. The reaction products were analyzed by RP-HPLC on a Gemini C18 column (4,6×250 mm, 5 µ, Phenomemex, Torrance, CA) by applying a gradient of water (A) and acetonitrile/methanol (6/4; v/v; B) at 0.5 ml/min. Elution started with 17% B for 10 min followed by an increase to 35% B within 60 min. Labeled sialic acids were monitored by fluorescence at λ_exc_ 373 nm and λ_em_ 448 nm.

## Results

### ES GNE−/− differentiating cells proliferate and differentiate slower than normal ES cells

Mouse ES cells can proliferate and differentiate in vitro. In certain culture conditions, these cells can differentiate spontaneously in to various lineages, in particular to neuronal cells, cardiac muscle cells and skeletal muscle cells [16]. Since we were interested in investigating the role of GNE in these tissues, we used mouse ES cells, wild type and GNE−/−, for our studies, and followed their differentiating course as described in the Methods section.

**Figure 1:**
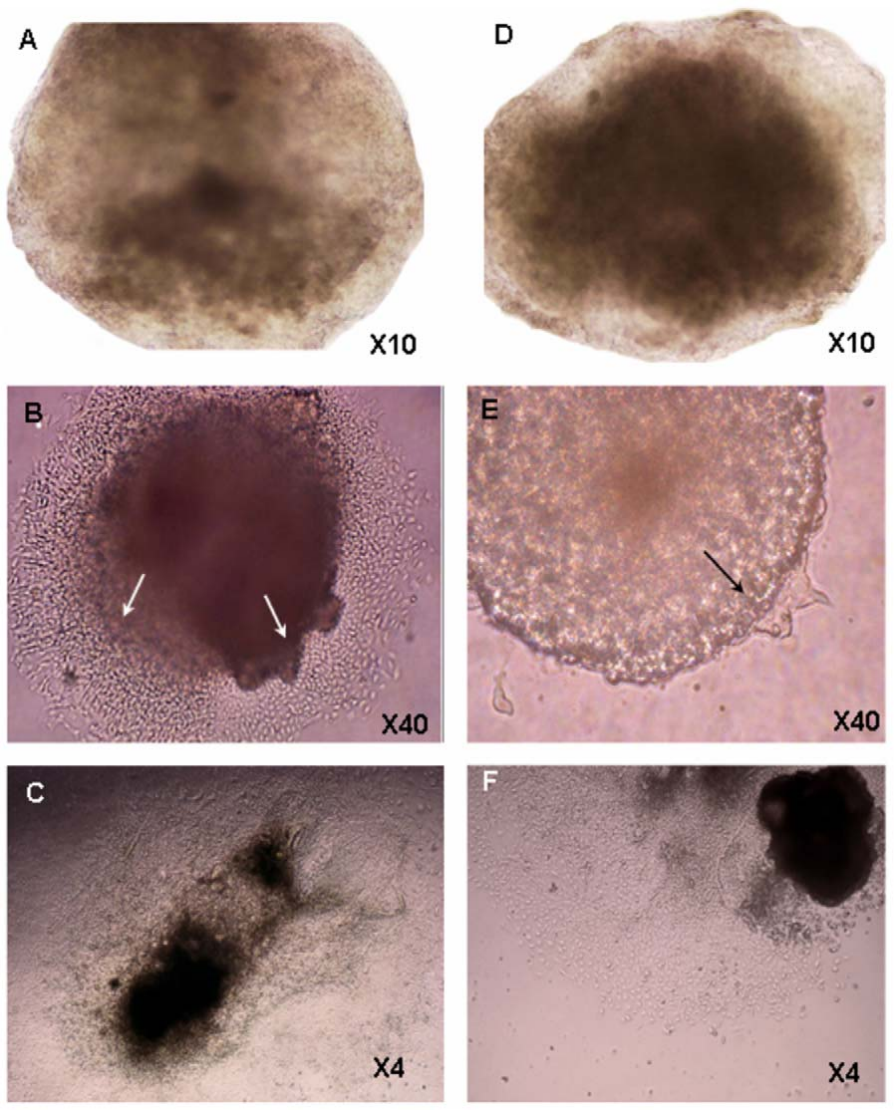
Morphology of ES early differentiating cells in culture. The morphology of GNE+/+ (A–C) and GNE−/− (D–F) ES cells was followed during differentiation in culture: there is no difference in recently formed EBs, at day 5 of differentiation (A and D). Later in differentiation, GNE−/− ES cells emerge (arrows) and proliferate slower (B and E show EBs 1 day after plating on gelatin coated plates-day 5+1; C and F , at day 5+6).

**Figure 2:**
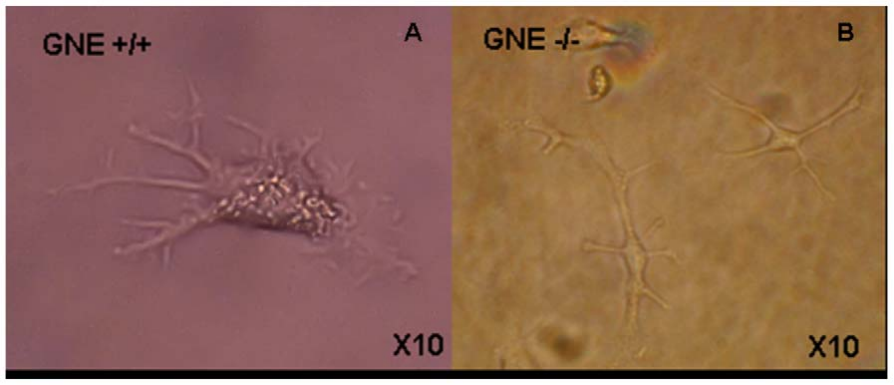
Neuronal lineage differentiation is delayed in GNE −/− ES cells. Well differentiated neuronal cells can be seen from day 5+12 in the GNE+/+ ES cells cultures (A), but only from day 5+40 in the GNE−/− ES cultures (B).

During cell culture expansion prior to the differentiation process, we noticed that the proliferation rate was about twice higher in GNE−/− cells than in normal ES (results not shown), in line with the observations reported by Weidemann et al [15] on these cells. The following examination of embryoid bodies (EBs), just before plating on tissue culture plates (day 5), showed some variation in the size and form of individual embryoid bodies, but with no consistent differences between GNE+/+ and GNE−/− EBs (Fig. 1A–D). Very soon after plating on gelatin coated tissue culture plates (Day 5+1), we could already detect differences between GNE +/+ and GNE−/− cells (Fig. 1B–E): in wild type cell cultures, new cells emerged from the EB borders, proliferated and were able to adhere and spread on the plate surface. In contrast, the number of cells emerging from the GNE−/− EBs was very limited. Five days later (Fig. 1C–F), at day 5+6 of differentiation, we could still observe a delay in the culture dynamics of the different cells: GNE+/+ cells covered already the entire culture surface, whereas the number of cells originating from the GNE−/− EB was significantly smaller, indicating either a much slower proliferation, adherence or/and migration capacity of GNE −/− cells compared to normal. Six days later (Day 5+12), GNE−/− cells also covered the entire culture surface, so that no proliferation rate difference could be detected between the 2 cultures from this time point (data not shown). Cell density decreased in the plate monolayer in direct correlation with the distance from the remaining EB aggregate (data not shown). In less dense regions, bigger cells could be seen to develop further (data not shown), such as cells displaying a morphology of neuronal cells which were observed relatively at distance from the EB itself, still only in the normal cell cultures (Fig. 2A). Most interestingly, at this time point, we could also see for the first time cells with cardiac muscle properties, that is which can beat (Video S1): in the more dense regions of both cultures we observed the beginning of fibers generation, that are able to contract. We could detect on average one beating focus per each EB, both in normal and in GNE −/− cell cultures.

**Figure 3:**
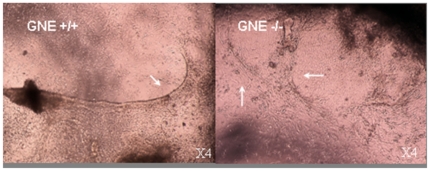
Cardiac fibers formation in ES cell cultures. Cardiac beating fibers (pointed by arrows) are present in both GNE+/+ and in GNE−/− ES cultures on day 5+20 after differentiation.

**Figure 4:**
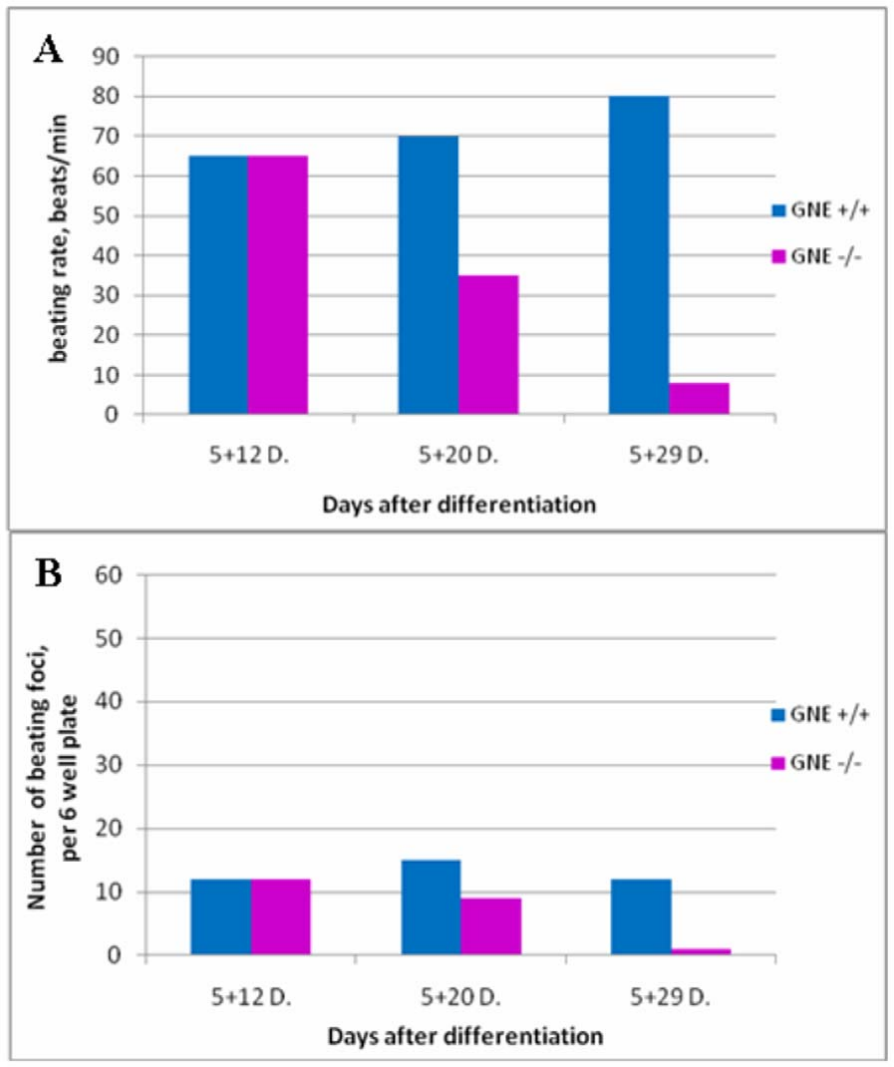
Cardiac muscle cells functionality. A –Beating rate of GNE+/+ (blue) and GNE−/− (pink) cells foci. There is a decrease in the beating pace of the GNE−/− cells foci with time. B – Number of beating areas per 6well plate. The amount of beating foci in the GNE−/− (pink) culture decreases with time, compared to the GNE+/+ (blue) cells.

At day 5+20, the formation of long fibers could be observed (Fig. 3) beating at a pace of 60–80 beats/min in normal cultures, as it was 8 days before, but already much slower in GNE−/− cultures (Fig. 4A). Also the number of beating foci decreased in the GNE−/− cultures (Fig. 4B). Ten days later, at day 5+29 and 5+30, the picture is quite similar to the previous time point: neuronal cells can be seen only in normal cultures, at the less dense regions, and cardiac fibers are still present mostly in the more dense regions of both cultures (data not shown). However, the number of beating foci kept decreasing in the GNE−/− cultures, as well as their beating pace, 1 to 5 beats/min compared to 50–80 for normal cells at the same time point. (Fig. 4A–B, Video S2). At this time point, we could detect the beginning of neuron like cells differentiation for the first time in the GNE −/− (Fig 2B) cultures, although they appear to develop much slower than in normal cultures and do not reach an advanced differentiation state as seen in GNE+/+ cultures, even much later in differentiation (at day 45).(Fig. 2B ).

### Marker expression analysis

Molecular markers representing the different differentiation stages of the different tissues were analyzed at various time points. cDNA was synthesized from RNA extracted from cell cultures of both GNE+/+ and GNE−/− origin, at several differentiation time points, and analyzed by PCR for the expression of various relevant genes: Oct4 as a marker of proliferating ES cells; NF-M (medium neurofilament) as a very early marker of neuronal precursor; cardiac β-MHC (cardiac myosin heavy chain beta) which is expressed mostly during embryonic development; Pax7 as a very early marker of skeletal muscle satellite cells commitment; MyoD, which is one of the earliest markers of myogenic commitment and is expressed in activated satellite cells, representing the beginning of the skeletal muscle differentiation process, and MyoR, a regulator of MyoD [18]. MyoR (for myogenic repressor), is expressed in undifferentiated myoblasts in culture and is down-regulated during differentiation. MyoR is also expressed specifically in the skeletal muscle lineage between days 10.5 and 16.5 of mouse embryogenesis and down-regulated thereafter during the period of secondary myogenesis. a role for MyoR as a lineage-restricted transcriptional repressor of the muscle differentiation program.

**Figure 5:**
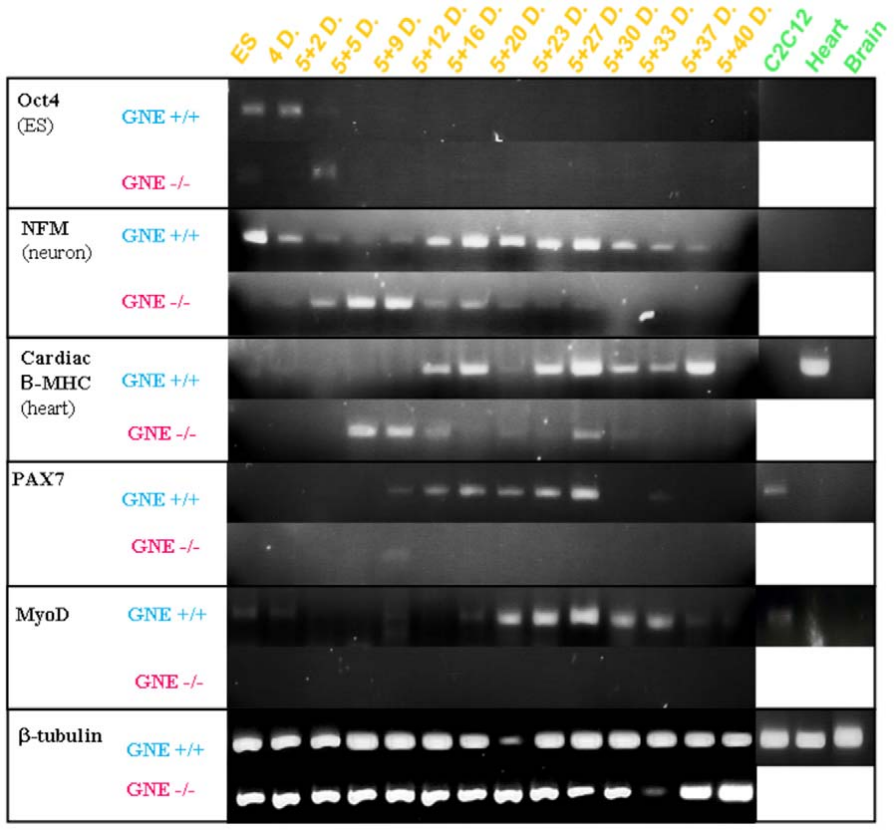
Differentiation expression pattern of ES cells in culture. GNE+/+ (blue) and GNE−/− (pink) ES cell cultures were analyzed for the RNA expression of several differentiation markers, by RT-PCR: Oct4, as a pluripotent ES marker; neuro-filament medium (NFM), as a neuronal marker; cardiac β-MyHC, as a cardiac cells marker;Pax7, as a skeletal muscle satellite cell marker and MyoD, as a skeletal muscle differentiating marker . β-tubulin was used as a mouse house keeping gene. As a positive control murine muscle C2C12 cells, as well as heart and brain tissues from wild type mouse were used (green panel).

**Figure 6:**
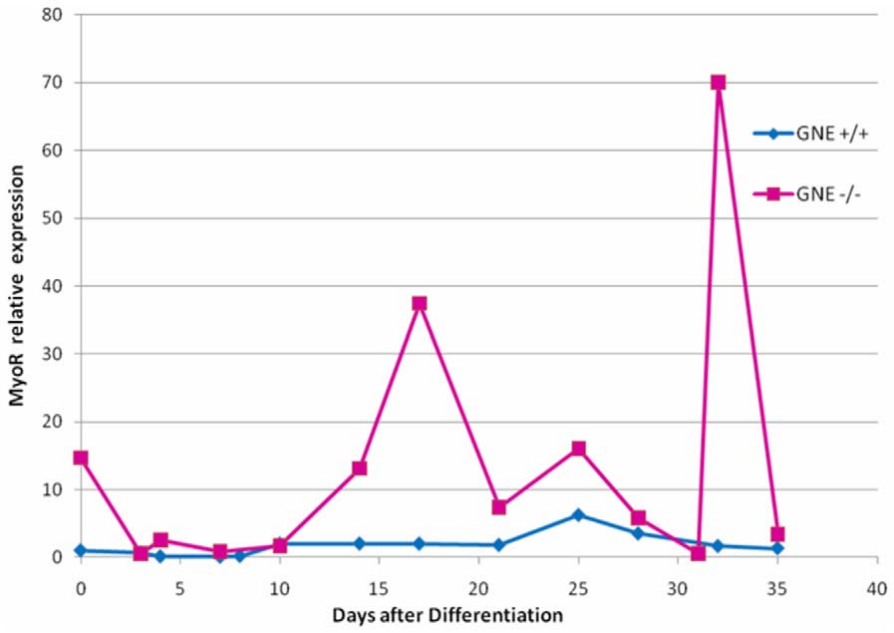
MyoR expression of ES cells in culture. GNE+/+ (blue) and GNE−/− (pink) ES cell cultures were analyzed for the RNA expression of MyoR by real time PCR at different timepoints. Expression is presented as relative quantity to MyoR mRNA of GNE+/+ ES at timepoint 0.

The results are showed in Figs 5 and 6. As expected (Fig. 5), Oct4 is expressed in normal and in GNE −/− ES cells at time 0 and at the very beginning of differentiation, up to day 5+5, and decays thereafter, in line with the beginning of the differentiation process. NFM is expressed also from the very beginning in normal ES cells, and its expression is maintained throughout the entire period examined. In GNE−/− cells, the expression of NFM is somewhat delayed and can be detected only around day 5+5. Also in correlation with the morphology, NFM expression decays with time and cannot be detected anymore from day 5+27. The cardiac β-MHC marker can be detected at day 5+12 in normal ES and even sooner in GNE −/− cells, but in contrast to its persistence in normal ES cells, its expression decreases with time in GNE −/− cells. This result is consistent with the morphological data reported above, which showed the decrease of functionality of GNE−/− cardiac cells in culture. As markers of skeletal muscle, we examined the expression of Pax7, and MyoD. We can see an almost complete lack of both markers in GNE−/− cells through the entire examination period, compared to normal ES cells which behave as expected: Pax7 begins to be expressed at day 5+9 and disappears around day 5+33, MyoD expression begins somewhat later, around day 5+12, while the differentiation process continues, and is continuously expressed till cells become senescent around day 5+40. By real time PCR, we have also analyzed the expression profile of MyoR a negative regulator of MyoD, which is relatively stable during all this time period in normal ES cells, but shows a much higher average expression in GNE−/− cells (Fig. 6). Altogether the results of the gene expression analysis strongly correlate with and support the morphological data.

### Immunohistochemistry

**Figure 7:**
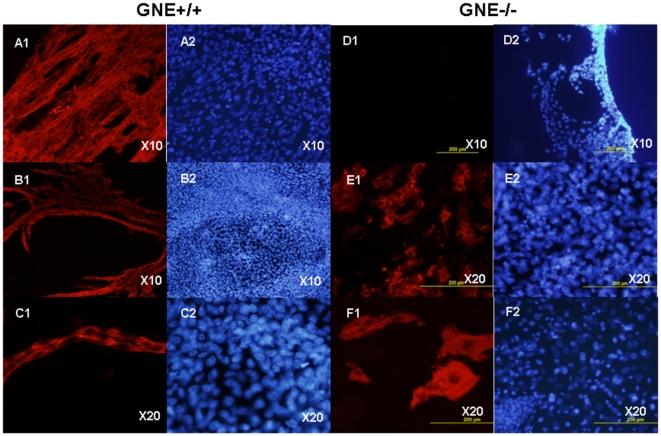
Impaired differentiation to skeletal muscle of GNE KO ES cells. GNE +/+ (A–C) and GNE−/− (D–F) cell cultures were stained with Myosin heavy chain (MyHC) antibody MF-20 (A1-F1) and Hoechst (A2-F2) at various stages of differentiation: A and D, day 5+12. B and E, day 5+20. C and F, day 5+35 of differentiation. Normal skeletal muscle structures are absent in the GNE −/− cultures, only very short myotubes can be seen.

Myosin heavy chain (MyHC), a marker of late differentiation of skeletal muscle, was examined by immunofluorescence. As seen in Fig. 7, MyHC can be detected in about 10% of the cultured normal ES cells but not before day 5+12 of differentiation, . Indeed the MyHC antigen is expressed in myotubes and multinuclear fibers. In contrast not a single MyHC positive cell could be detected in GNE−/− cultures till day 5+20, but even then the positive stained cells had a morphology which is very different from that of GNE+/+ cells: in cultures derived from normal ES cells, skeletal muscle cells are in a advanced differentiated state represented by very long fused myotubes generating large organized muscle fibers. In contrast, GNE−/− MyHC positive cells consist of single cells more elongated or of very short multinucleated shapes. At day 5+35, normal cells begin to show morphological signs of senescence, but we can still find some fully developed long multinucleated fibers intact, whereas GNE−/− cultures are completely devoided of muscle fibers and only very few myotubes can be observed. These results indicate that skeletal muscle development is severely impaired in GNE−/− mouse ES cells.

### Sialic acid levels measurements

Sialic acid analysis of the membrane glycoprotein fractions of 300,000 cells showed the presence of Neu5Ac as well as trace amounts of Neu5Gc in both samples, the analysis was performed in duplicate and the average values are presented in Table 1. WT ES cells contained approximately twice more Neu5Ac than the GNE KO ES cells.

**Table 1:** Sialic acid determination (in picomoles, pmol) of GNE+/+ and GNE−/− ES cells at timepoint 0, using DMB-HPLC.

ES cells	Neu5Ac(pmol)	Neu5Gc(pmol)
GNE KO	160	19
GNE wt	314	2

## Discussion

In the context of the recessive disease Hereditary Inclusion Body Myopathy (HIBM), caused by missense mutations in GNE, we were interested in elucidating possible roles of GNE specifically in skeletal muscle. The role of GNE in the sialic acid pathway has been well established, as well as the importance of sialylation in many diverse biological pathways mostly involving cell-cell recognition mechanisms, in particular in brain development. In an attempt to clarify whether GNE is directly involved in muscle development, we have take advantage of the availability of GNE KO ES cells derived from homozygous GNE KO embryos at day E3.5 [15] and have compared their developing profile to GNE homozygous normal ES cells. It has been shown previously that spontaneous differentiation of mouse ES cells into various cell types including cardiac and skeletal muscle as well as neuronal cells is achieved by culturing the cells as aggregates (Embryoid bodies, EBs) in differentiation medium with 1% DMSO, followed by tissue culture plating [16].

In this study, we could recapitulate the time course development of normal ES cells as reported before [16] and we found a strong correlation between the morphology and the molecular data presented.

As expected, we found that GNE has a great impact in the development of neural cells; indeed the role of GNE and sialic acid in brain development and plasticity has been well established. It has been shown that the polysialic acid chain bound to the neural cell adhesion molecule (NCAM) is responsible for the neural plasticity during fetal development and in the brain of the adult [19]–[21]. Also gangliosides have been found to strongly contribute to brain development [22]. Our results confirm these findings. To date, no data was available for assessing the role of GNE in muscle development. The present findings suggest a very close to normal development of cardiac cells which are even functional at the very first stages of development, as determined by their morphology, the expression of the relevant molecular markers and their beating pace, followed by a sharp loss of this functional property early after with the appearance of senescence and degeneration symptoms. The fact that at the beginning of differentiation (day 5+12) we cannot detect differences in the number and the pace of the beating cells appearing in both normal and GNE KO ES cells indicates that GNE is not necessary for the development of individual functional cells; however, their inability to generate fibers and their functional decay and following degradation already at day 5+20 post differentiation could indicate a defect in organization capacity, expressed by the inability of the cells to form more sophisticated structures. In this context it could be possible that GNE KO mice die at day E8.5 because of defective cardiac function.

Skeletal muscle development in GNE−/− ES cultures seems to be affected even earlier. Normal ES cells can differentiate to satellite skeletal muscle cells, as revealed by the expression of Pax7, MyoD MyHC and MyoR in GNE+/+ cell cultures. Although we could not detect clearly cells with skeletal muscle morphology in either the GNE+/+ or the GNE−/− cultures, probably because the density of the cultures at this stage (only 5–15% of the stem cells plated is expected to differentiate to skeletal muscle cells [16], the expression analysis of Pax7 and MyoD genes revealed very scarce muscle satellite cells and subsequently an almost complete lack of cells committed to skeletal muscle differentiation among GNE−/− cells compared to GNE+/+ cells, as MyHC staining emphasized the lack of myotubes in these GNE KO cultures. Further, MyoR expression was relatively high in these cells. Musculin/MyoR is a negative transcription regulator of satellite cell differentiation [18] and its inverse correlation to MyoD expression could indeed support a differentiation defect in GNE−/− ES differentiating cells. Skeletal muscle cell elongation and myotube formation seems to be fully absent in our GNE−/− cultures, although some cells succeeded in reaching few more differentiated stages, as detected in older cultures by very few and very short structures expressing the MyHC protein. Thus, this data strongly supports a very early and severe defect in cardiac and skeletal muscle development of GNE−/− cells.

Our studies clearly show the importance of GNE in the development of embryonic stem cells derived muscle tissue progenitors , both skeletal and cardiac. Indeed fiber generation requires the concurrent recruitment of many biological processes which involve a high number of proteins in the mature fiber for the common and complex task of sarcomere organization. To note, previous studies have point to a novel possible role for GNE in muscle filament structures by proteomics analysis [23], and by its capacity to bind to actinin1 next to the Z line as well as at the M line [24]. The results of the present study strongly supports the involvement of GNE in a novel function in sarcomere formation. The question raises whether the dramatic effects on muscle development are a direct consequence of the lack of this protein or whether they are mediated by a lack of the sole known product resulting from the enzymatic activity of GNE, sialic acid. Indeed, it cannot be ruled out that sialic acid is the basic explanation of the phenomenom, however the sialic content of the ES GNE−/− cells is about half of the GNE+/+ cells; since sialic acid shows a broad normal physiological range in human [10], [11], and since heterozygous GNE +/− mice are healthy [14], it is highly unlikely that the dramatic effects reported in our study could be accounted for a relatively low reduction of the sialic acid level in the cells. Therefore it is very likely that at least part of the cellular changes seen in GNE−/− ES cells compared to GNE+/+ ES cells occur as a direct result of a GNE function(s) other than sialic acid synthesis.

Interestingly, we have observed an increase of NeuGc in GNE−/− ES cells. The knockout of GNE results in the inactivation of the synthesis of sialic acids using the *de novo* pathway. In this situation, cells incorporate sialic acids stemming from the cell culture medium into lysosomes where they are released from exogenous glycoconjugates [25]. Free sialic acids are then activated by CMP-Sia synthases (CMAS) and transferred to glycoconjugates. Nystedt and coworkers [26] recently showed the upregulation of CMP-Neu5Ac hydroxylase (CMAH), which converts CMP-Neu5Ac into CMP-Neu5Gc, in human stem cells. Therefore, the increase of NeuGc in ES GNE−/− cells compared to GNE+/+ cells might be explained by the upregulation of CMAH.

Although our results do not clarify the function of GNE in later stages of muscle development, accumulating evidence is pointing to novel GNE functions in addition to its well established and investigated role as a bifunctional key enzyme in the sialic acid pathway. First, its ability to regulate sialyl transferases [27]; second, its binding capacities to α-actinin1 in skeletal muscle [24], and very recently, its involvement in neurite differentiation [28] and in proliferation of ES cells [15]. Also the present studies show that the proliferation, adherence and/or migration and tissue organization capacities of GNE−/− ES cells are defective at their very first stages of differentiation.

HIBM is a slowly progressive, adult onset disorder, characterized by skeletal muscle atrophy solely. The disease results from missense mutations in GNE, and the entire mutated protein is present at normal levels and at the appropriate location in patients'skeletal muscles [8], [9]. The first hypothesis considered in HIBM pathogenesis is a change in the sialylation pattern potentially caused by a defective GNE activity. However, investigations of the GNE enzymatic functions showed that the extent of the activity reduction in lymphocytes, myoblasts and myotubes of HIBM patients varied only around 30%, [10], [11] making it difficult to explain how such partial enzymatic activity reduction could lead to markedly reduced sialic acid production: indeed it has been shown that in normal tissue GNE activity consists of only 5% of its potential [27] and theoretically this could be augmented in HIBM by compensatory activation; furthermore, additional kinases present in the cell, such as N acetyl glucosamine kinase (NAGK), are capable to use N acetyl mannosamine as a substrate [29] and therefore could compensate for the partial defective function of the kinase activity of GNE, at least in HIBM cells carrying the homozygous M712T mutation at the kinase domain, which is the common Middle Eastern mutation. Further, HIBM is an adult onset disorder, thus excluding the possibility of early important developmental defects, which would be expected as the result of the involvement of sialic acid dysfunction, which most likely would present a more severe and early phenotype.

Various directions have been undertaken to try and unravel novel potential functions for GNE, which could lead to better insights into the pathogenesis of HIBM. Among those, satellite cell dysfunction [30], modulatory effects on other biochemical pathways [27], a yet undetermined nuclear activity [9], [31] and recently identified protein partners [24], [32] are to be noted. Certainly the essential role of GNE involving sarcomere organization, maintenance and robustness in the development of cardiac and skeletal muscle showed here, will bring novel understandings in muscle function mechanisms.

## Supporting Information

Video S1 Functional cardiac cells in cultures of normal (N ES) and GNE KO ES (KO ES) cells at day 5+12 post differentiation. Both cell types contract at similar pace.
(MPG)

Video S2 Functional cardiac cells in cultures of normal (N ES) and GNE KO ES (KO ES) cultures at day 5+30 post differentiation. Normal cells contract at the same pace than at day 5+12, in contrast GNE KO cells contractions are much slower.
(MPG)

Table S1 Primer sequences used for analysis of expression markers.
(DOC)
