# Supplementary material for: GNE Is Involved in the Early Development of Skeletal and Cardiac Muscle
Source: PLoS One. 2011 Jun 24;6(6):e21389. doi: 10.1371/journal.pone.0021389 (PMC3123316; doi:10.1371/journal.pone.0021389)
Supplement: Table S1 — Primer sequences used for analysis of expression markers. (DOC) [file pone.0021389.s003.doc]

Table S1. Primer sequences

| Primer sequence (5’ - 3’) | | Gene |
| --- | --- | --- |
| Reverse | Forward |  |
| tcactgtgcctgaacttacc | ggaacatagccgtaaactgc | β tubulin |
| ctgtagggagggcttcgggcactt | ctgagggccaggcaggagcacgag | Oct 4 |
| gcctcgactttggtttctg | gggaaccaagtgggaaatgg | NFM |
| ctgtcctctgtatggcatcc | gatctctcagctaacccgagg | β MHC |
| gttagctcctgcctgcttacg | gagttcgattagccgagtgc | Pax 7 |
| gctctgatggcatgatggattacagcg | atgctggacaggcagtcgaggc | MyoD |
| ccagagaccacgaatggcca | accgcracgaggacagctat | MyoR |
| cacccttcaagtgggcccc | gcaaattcaacggcacagt | Mouse GAPDH |
